# A Hypoxia Gene-Based Signature to Predict the Survival and Affect the Tumor Immune Microenvironment of Osteosarcoma in Children

**DOI:** 10.1155/2021/5523832

**Published:** 2021-07-15

**Authors:** Feng Jiang, Xiao-Lin Miao, Xiao-Tian Zhang, Feng Yan, Yan Mao, Chu-Yan Wu, Guo-Ping Zhou

**Affiliations:** ^1^Department of Pediatrics, The First Affiliated Hospital of Nanjing Medical University, Nanjing 210029, China; ^2^Department of Neonatology, Obstetrics and Gynecology Hospital of Fudan University, Shanghai 200011, China; ^3^Department of Rehabilitation Medicine, The First Affiliated Hospital of Nanjing Medical University, Nanjing 210029, China

## Abstract

Osteosarcoma is a quickly developing, malignant cancer of the bone, which is associated with a bad prognosis. In osteosarcoma, hypoxia promotes the malignant phenotype, which results in a cascade of immunosuppressive processes, poor prognosis, and a high risk of metastasis. Nonetheless, additional methodologies for the study of hyperoxia in the tumor microenvironment also need more analysis. We obtained 88 children patients with osteosarcoma from the Therapeutically Applicable Research to Generate Effective Treatment (TARGET) database and 53 children patients with RNA sequence and clinicopathological data from the Gene Expression Omnibus (GEO). We developed a four-gene signature related to hypoxia to reflect the immune microenvironment in osteosarcoma that predicts survival. A high-risk score indicated a poor prognosis and immunosuppressive microenvironment. The presence of the four-gene signature related to hypoxia was correlated with clinical and molecular features and was an important prognostic predictor for pediatric osteosarcoma patients. In summary, we established and validated a four-gene signature related to hypoxia to forecast recovery and presented an independent prognostic predictor representing overall immune response strength within the osteosarcoma microenvironment.

## 1. Introduction

Osteosarcomas are primary malignant tumors of the bone that are defined by the malignant cells' production of osteoid or immature bone. Osteosarcomas are rare; in the United States, around 750 to 900 cases are identified each year, with 400 cases occurring in children and adolescents under the age of 20 [1, 2]. It is most likely to develop in children and teenagers [3]. Osteosarcoma is particularly malignant, and it grows across the body and metastasizes to the lungs [4, 5]. The 5-year rate of patients with localized osteosarcoma is 80%, while that of patients with metastatic osteosarcoma is 15-30% [6]. For those with metastatic osteosarcoma, the survival rate is very low [7]. The advances in surgery, chemotherapy, and radiation therapy are highly significant since they can decrease the occurrence of lung metastasis and increase the long-term survival in patients with osteosarcoma [8]. The poor prognosis is due to difficulty in early detection, high incidence of metastasis, and relapse. The process by which the tumor grows was not completely elucidated.

Hypoxia is a characteristic that happens when the intake of oxygen is too inadequate to support the growth rate of cancerous tumor cells [9]. Current reports have shown the crucial effect of hypoxia on cell proliferation, tumor growth, and cell differentiation [10]. Bone is one of the most vulnerable organs to hypoxia and has been shown to play a major role in metastasis, weak prognosis, and radiation tolerance of osteosarcoma [11, 12]. Nonetheless, the regulatory structure remains uncertain. The study focuses on the tumor microenvironment and immune cells that are active in tumor progression. Studies have shown that the hypoxic microenvironment can encourage the mobilization of innate immune cells in tumors and reduce adaptive immunity in the tumor microenvironment [13, 14]. As a result of the limited literature on the relationship between hypoxia and immunity, new therapeutic techniques are required.

Approximately one-third of ongoing osteosarcoma clinical studies (as reported by ClinicalTrials.gov) use targeted medications that target all parts of cellular functions. It is obvious that targeted therapy plays a critical role in the treatment of osteosarcoma. In an SJSA-1 osteosarcoma xenograft model, Chessari et al. discovered a potent isoindolinone inhibitor of the MDM2-p53 interaction, which demonstrated critical anticancer efficacy [15]. Trametinib and trastuzumab, two MEK inhibitors, were investigated in a phase I/II clinical study and a phase II clinical study for osteosarcoma or recurrent osteosarcoma, respectively [16]. However, there is still a disconnect between what we know about osteosarcoma biology and how it affects patients. In this research, we aimed at hypoxia-related genes in osteosarcoma using the TARGET and GEO databases to create a predictive model for predicting its immune microenvironment in children with overall survival (OS). This study may play a major role in aiding medical professionals in making critical clinical choices.

## 2. Materials and Methods

### 2.1. Data Selection and Analysis

A total of 88 osteosarcoma samples with RNA-Seq data and clinical details were retrieved from the TARGET (https://ocg.cancer.gov/programs/target) database. The GSE21257 collection containing 53 samples was downloaded from GEO (https://www.ncbi.nlm.gov/geo) as a validation set. The clinical features of the samples are provided in Table S1.

### 2.2. The Integration of Immune Cell Types

CIBERSORT, a method developed by Newman et al. (https://cibersort.stanford.edu/), is a process for estimating the relative abundances of types of cells in a mixed population of cells [17]. We calculated how proportionally immune cell types were distributed across the low and high hypoxia risk groups. The score given to each immune cell class in each study is equivalent to 1.

### 2.3. The Development of a Hypoxia Risk Model

The genes related to hypoxia statistically relevant in univariate analysis were then shown to be significant in multivariate analysis which produced the risk score formula [18], which is risk score = *β*_1_*X*_1_ + *β*_2_*X*_2_ + ⋯+*β*_*n*_*X*_*n*_, where *X*_1_, *X*_2_, ⋯, *X*_*n*_ were the level of the considered predictors and the “*β*” parameters were respective regression coefficients.

### 2.4. Survival Analysis

Overall survival between the high and low hypoxia risk groups was evaluated by a log-rank test in R software (version 3.8.2). Univariate analysis was carried to identify prognosis predictors, and multivariate analysis was performed to assess if the score for risk is predictive of survival. We gathered data to determine the model's capability to assess the outcomes of patients.

### 2.5. GSEA

The gene set enrichment study was conducted to decide whether there was a substantial variation in the genes that are expressed between the high and low-risk classes of the MSigDB (c5.bp.v7.0.symbols.gmt; h.all.v7.0.cymbols.gmt) [19]. The gene set permutations were tested 1,000 times to illustrate its capacity to function consistently. The phenotype label was used to forecast adverse events.

### 2.6. PPI Network

The STRING database was used in building a PPI network. Cytoscape was also used to measure the node degree of the PPI [20], which is the number of relationships to filter the key genes.

## 3. Results

### 3.1. Application of a Hypoxia Risk Signature to Assess Prognosis of Children with Osteosarcoma

The genes involved in hypoxic responses were derived through the GSEA study (hallmark; hypoxia), which was performed under conditions with hypoxia. To better explain the molecular interactions that arise while a stressed cell is deoxygenated, we performed PPI studies utilizing the STRING database (http://string-db.org). Figure S1 charts the top 150 genes which have the highest degrees involved in the reaction to hypoxia.

**Figure 1:**
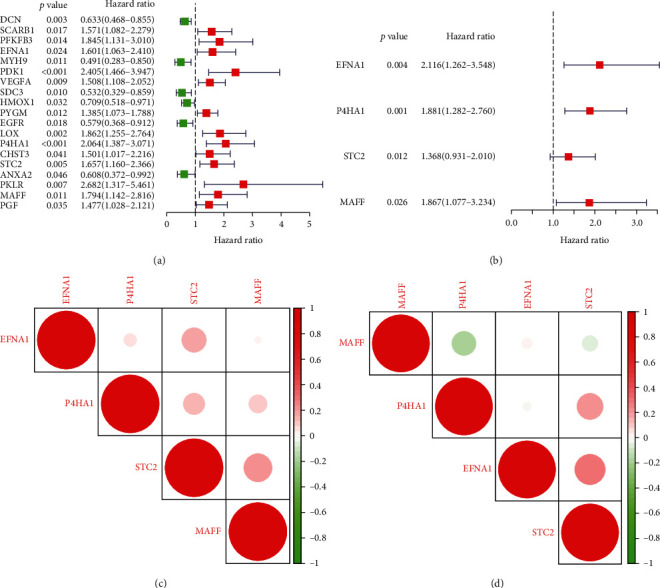
Characterizing hypoxia risk signatures in osteosarcoma to assess mortality and clinical outcomes: (a, b) development of a hypoxia susceptibility signature to assess children with osteosarcoma prognosis through univariate and multivariate Cox analyses; (c, d) Spearman relation study of a four-gene signature in TARGET and GEO databases.

To assess the prognosis for osteosarcoma patients based on an analysis of gene expression, by using univariate and multivariate regression, we conducted the list of top 100 genes in the TARGET training set. The researchers observed that 19 genes related to hypoxia were correlated with children's OS (Figure 1(a)). Four hypoxia-related genes were selected to create a predictive model. These genes were EFNA1, P4HA1, STC2, and MAFF (Figure 1(b)). The risk score formula was constructed as

(1) $$\begin{array}{c} \text{risk score}=\left(0.74\times \text{EFNA}1+\left(0.63\times \text{P}4\text{HA}1\right)+\left(0.31\times \text{STC}2\right)+\left(0.62\times \text{MAFF}\right).\right. \end{array}$$

All four genes were shown to be linked to each other in association with other genes (Figures 1(c) and 1(d)).

### 3.2. The Predictive Consequences of a Hypoxia-Induced Signature in Osteosarcoma Patients

**Figure 2:**
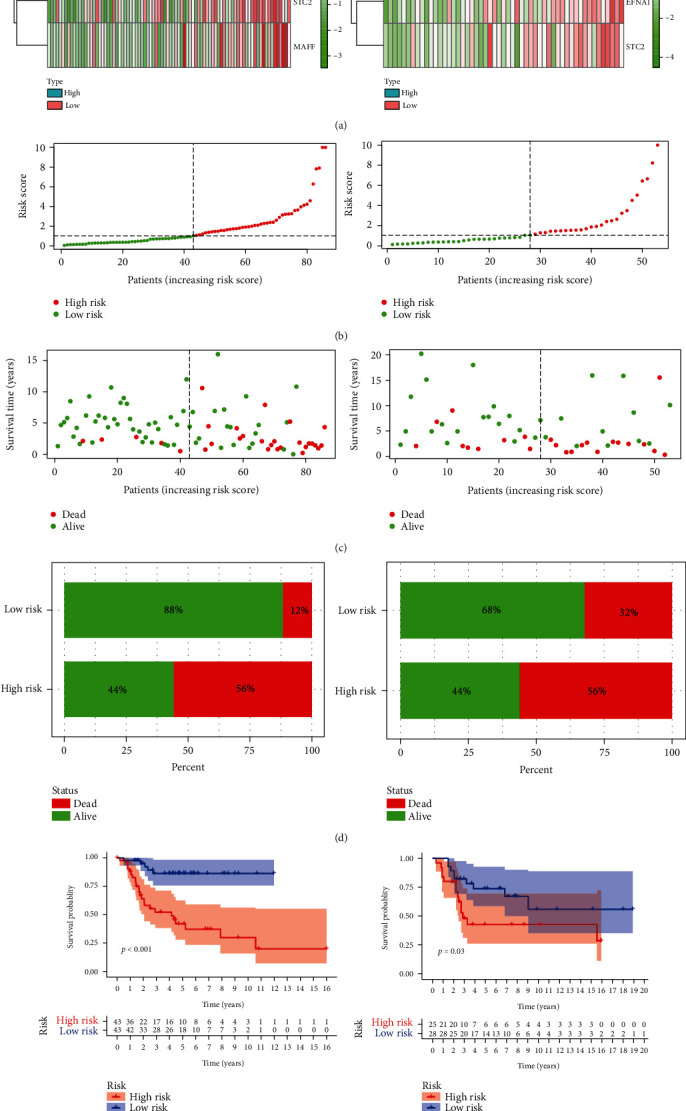
Prognostic importance of the four-gene signature in osteosarcomas: (a) a heat map displaying four-gene expression patterns in two groups from TARGET and GEO databases; (b, c) the patient status distributed in the two risk groups; (d) the death ratios for the two groups; (e) the survival curves for patients depending on risk.

Due to the effect on tumor cells under hypoxia, we also investigated whether the hypoxia signature has prognostic significance. Using the scores, we determined the rank and then grouped the patients into high- and low-risk categories depending on the median ranking (Figure 2(b)).  Figure 2(a) reveals that the expressions of EFNA1, P4HA1, STC2, and MAFF increased as risk scores increased in the TARGET and GEO datasets, which means that children with elevated risk appear to establish a hypoxic microenvironment. Our studies suggest that the people who are in the high-risk group had a consistently higher risk of death (Figures 2(c) and 2(d)). In addition, Kaplan-Meier research was conducted to determine the predictive importance of the hypoxia-related signature in children with osteosarcoma. The figure depicts that a high risk of hypoxia was correlated with poor overall survival in the TARGET cohort, which is confirmed by GEO results.

### 3.3. Gene Expression in reaction to Hypoxia Is Associated with Clinicopathological Features in Osteosarcoma in Children

**Figure 3:**
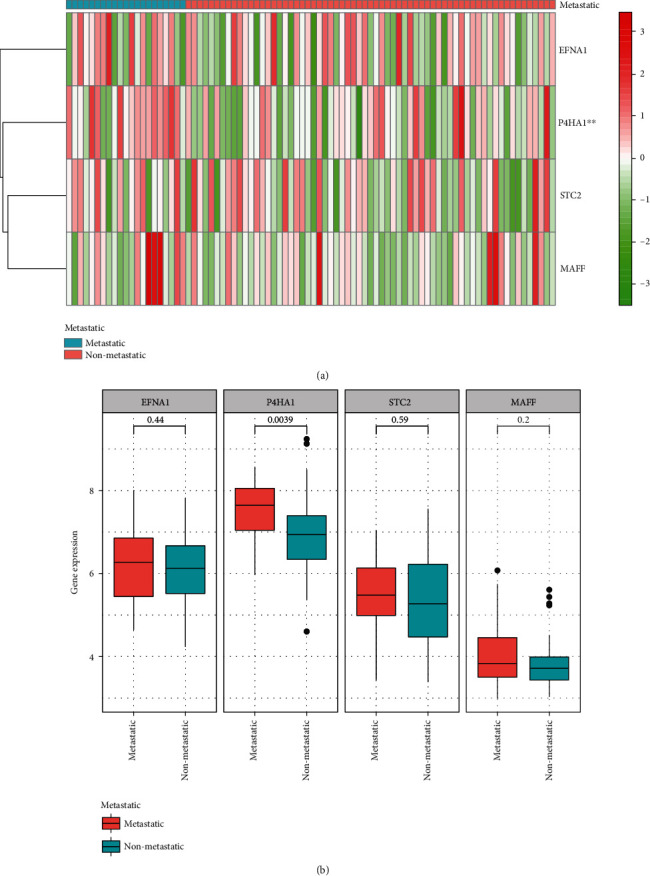
Hypoxia-associated gene is associated with clinicopathological characteristics of osteosarcoma in children: (a) a heat map displaying hypoxia gene expression profiles in TARGET database of metastatic and nonmetastatic patients; (b) the expression levels of four genes in osteosarcoma patients.

In order to explore the biological functions of hypoxia in tumorigenesis and development, we examined the relationships between four genes linked to hypoxia and certain characteristics of children with osteosarcoma, including if they had metastasized. Gene expression values and metastasis status were compared (Figure 3(a)), showing that gene P4HA1 increased in the metastasis group (Figure 3(b)).

### 3.4. The Hypoxia Risk Signature Is an Important Instrument for Prognosis Evaluation

**Figure 4:**
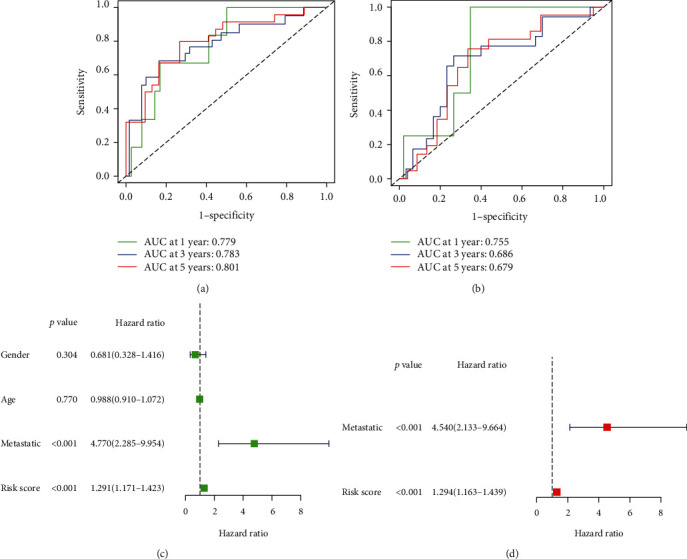
Prognostic importance of the 4-gene signature in osteosarcomas: (a, b) the predictive strength of the four-gene signature was measured with Kaplan-Meier survival curves for contrast; (c, d) univariate and multivariate Cox studies assessing the independent prognostic significance of the four-gene signature in terms of overall survival in pediatric osteosarcoma patients in the TARGET dataset.

The risk signature was modeled using the ROC (receiver operating characteristic) curve method on survival analysis from the TARGET and GEO datasets. AUC was 0.779 at one year, 0.783 at three years, and 0.801 at five years, suggesting a strong predictive value (Figure 4(a)). This research was tested using GEO criteria (Figure 4(b)).

Univariate and multivariate Cox survival analyses were done to assess the prognostic importance of a hypoxia signature on the survival of patients with osteosarcoma. The univariate study found that a high risk for hypoxia was linked to low survival (Figure 4(c)). Other factors such as metastatic status were also related to poor survival. Multivariate analysis found that a high risk of hypoxia for children with osteosarcoma was substantially correlated with a significantly lower overall survival of children with osteosarcoma.

### 3.5. Gene Set Enrichment Analysis

**Figure 5:**
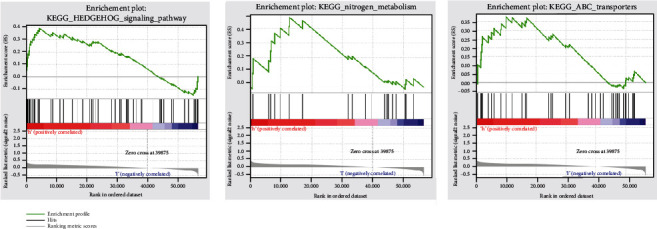
GSEA shows enrichment between the two risk categories.

To further validate that these elevated risk groups had altered signaling mechanisms, we evaluated the GSEA comparing the high vs. low hypoxia groups. Gene sets were elevated in the high-risk group in the TARGET database, such as hedgehog signaling pathway, nitrogen metabolism, and abc transporters (Figure 5, Table S2).

### 3.6. Immune Landscape in Children with Osteosarcoma between High and Low Hypoxia Risk

Accumulating evidence shows that tumor microenvironments can protect tumors from immune defenses by promoting immune escape and inhibiting immune effector cells. Here, we studied whether a hypoxic hypoxia signature of an immune microenvironment could be evaluated.

**Figure 6:**
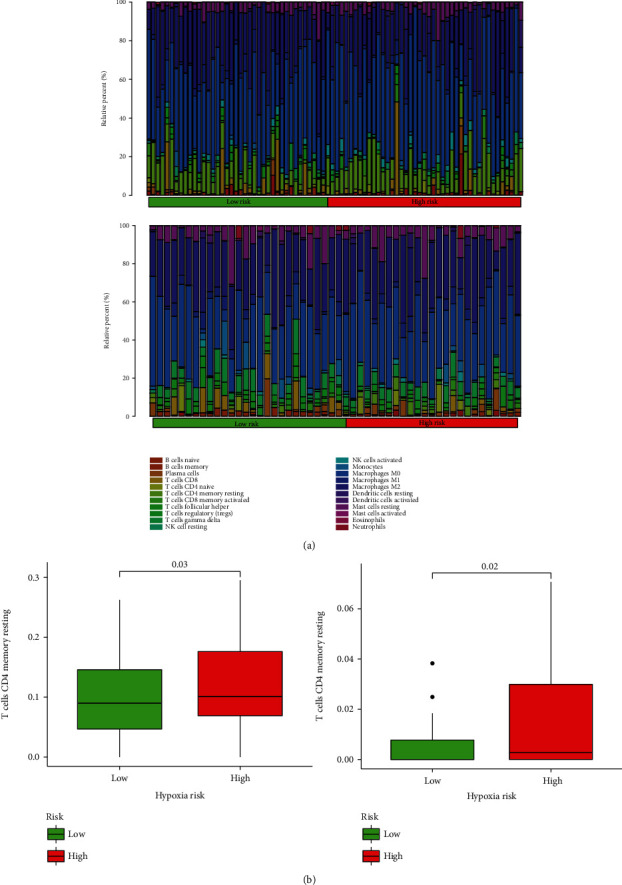
The immune landscape of children with osteosarcoma in two groups: (a) the relative frequency of different immune cells between patients in two groups with osteosarcoma; (b) box plots indicating different immune cells in children with osteosarcoma between two groups.

CIBERSORT was used to identify variations in the immune penetration of 22 different immune cell forms between low- and high-risk children with osteosarcoma. In Figure 6(a), the outcomes of patients in the TARGET database and GEO dataset are summarized. Patients with elevated levels of hypoxia risk have a significantly higher number of immune suppression and immunosuppressive cells (Figure 6(b)). The microenvironment can be influenced by the immunosuppressive cells.

### 3.7. An Elevated Hypoxia Risk Suggests a Suppressive Immunologic Environment

**Figure 7:**
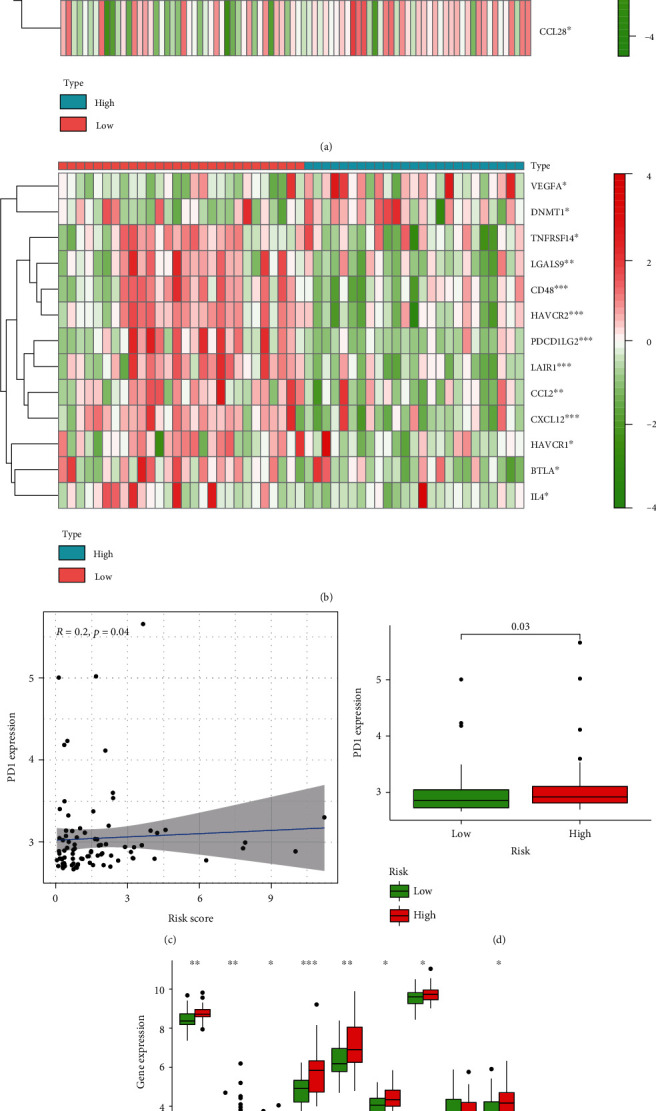
High-risk score suggests a microenvironment with immunosuppression: (a) a heat map of the gene profiles participating in cancer/immune system cycle in two groups in TARGET and GEO databases; (b) association between PD1 expression and risk score; (c) correlation between PD1 and risk score; (d) PD1 level in two groups; (e) tumor immune cytokine expression in two groups.

The cancer-immunity cycle helps explain how cancer and immunotherapy function. The research examines how cancer cells are destroyed by the immune system: cancer cell antagonism, cancer antigen introduction, priming and activating, trafficking of T cells to tumors, intratumoral T cell aggregation, cancer cell recognition by T cells, and cancer cell death [21]. The study analyzes the expression of various genes in the high- and low-risk groups. Gene details are downloaded from the TTI database [22] (http://biocc.hrbmu.edu.cn/TIP/index.jsp). As shown in Figures 7(a) and 7(b), immune genes that lead to a reduced chance of contracting cancer were more highly expressed in the high-risk group, suggesting low activity of these mechanisms in these patients.

Focused on previous studies showing that immune checkpoint molecules can be upregulated in reaction to hypoxia [23], we conducted an analysis of the expression level of molecules in the low- and high-risk groups in the TARGET dataset. We found that PD1, which is thought to be upregulated in hypoxic environments, has high expression in the high-risk group (Figures 7(c) and 7(d)). Certain cytokines were elevated in high-risk groups attributed to hypoxia (Figure 7(e)).

## 4. Discussion

The treatment of osteosarcoma and the prognosis have not improved significantly over the past three decades. In recent decades, high-throughput sequencing has become an essential instrument in biomedical science and is used for prognosis forecasting, cancer recurrence monitoring, and clinical stratification since it will be important to apply to osteosarcoma and identify possible key targets for therapy.

Malignant tumors often exhibit dysregulation in cellular oxygen metabolism. It was shown in various studies that hypoxia played a role in the development of this cancer [24]. Beyond the role of oxygen supply, the precise role of hypoxia in osteosarcoma remains uncertain. In this study, we explored the role of four genes (EFNA1, P4HA1, STC2, and MAFF) profiled in a previous study on osteosarcoma. EFNA1 is a transmembrane protein, and EFNA11 expression is upregulated in a number of tumor cells, including gastric cancer, colorectal cancer, and hepatocellular cancer [25]. Large levels of an enzyme linked with cancer survivability have a weak prognosis for cancer patients. P4HA1 is an essential enzyme in hemoglobin synthesis [26]. Previously, it has been shown that P4HA1 stabilizes hypoxia-inducible factor 1*α* (HIF1*α*) by modulating the levels of its glycolytic substrates, *α*-ketoglutarate, and succinate, thus mediating cellular transformation of cancer cells [27]. The STC2 protein encodes a secreted homodimer and is expressed in a broad range of tissues. The protein has autocrine and/or paracrine roles [28]. Studies have demonstrated that STC2 is oncogenic in several forms of cancer. The aberrant STC2 expression has significant consequences for the prediction, incidence, metastasis, and prognosis of different cancers, including various cancers of the liver, colon, and prostate [29]. MAFF is a part of the essential leucine zipper (bZIP) transcription factor Maf family and plays a key role in numerous physiological and pathological processes, including signal transduction, hematopoiesis, central nervous system activity, and tumorigenesis [30, 31]. With the four-gene signatures, it was seen that this was an independent factor that affects the prognosis of osteosarcoma and that the model had a stronger effect in forecasting osteosarcoma.

Our GSEA revealed that hedgehog signaling pathway, nitrogen metabolism, and abc transporters were enriched in the high-risk group in the TARGET database. Onishi et al. reported that hypoxia can activate the hedgehog signaling pathway in a ligand-independent manner by upregulation of Smo transcription in pancreatic cancer [32]. Kodama et al. found that accumulation in glutamine nitrogen metabolism can contribute to the malignant progression of tumors [33]. abc transporters also have been considered for their contribution to cancer cell biology recently, and they may play a potential role in cancer development [34].

Many studies have been conducted investigating the processes by which hypoxia controls the immune response in tumor cells [35, 36]. The lack of oxygen will negatively affect the differentiation and work of immune cells and their subsequent cytokine development [37]. The immune system can detect and eliminate tumor cells [38, 39]. Nonetheless, the tumor microenvironment could interact with this immunotherapy reaction by blocking various checkpoints through which the therapy operates [40]. Regulation of NK cells and tumor antigen-specific T cells is a key step that must take place for an antitumor immune response [41]. Hypoxia prevents T and NK cell development and activation in animal models [42]. Our analysis showed that children with osteosarcoma at high risk of experiencing hypoxia had an elevated number of CD4 cells, suggesting an immunosuppressive disorder.

Cytokines have the capacity to control immune responses. Immune cell exhaustion is one of the main factors supporting tumor progression [43]. The hypoxic microenvironment of tumors is associated with VEGF and stimulates tumor development [44]. CCL28, a crucial mediator between oncogenic *β*-catenin signaling and the stomach tumor microenvironment, is shown to have an integral part of cancer development by either facilitating cancer cell proliferation or metastasis or by forming the tumor immune microenvironment [45]. In our research, the immune system is weakened in the high hypoxia community, contributing to greater suppression of the immune system. Immune checkpoint proteins safeguard against cancer and support tumor immunosuppression. Tumors defend themselves using immunotherapy modalities and clinical goals (e.g., CTLA-4, PD-L1, and PD1) [46, 47]. In our research, immunosuppressive cytokines and PD1 have high expressions in participants with higher risk of developing hypoxia.

This is the first analysis to construct and evaluate a risk model related to hypoxia based on four genes and then use it as a prognostic factor for osteosarcoma patients. Our results indicate that hypoxia triggers immune cell death in osteosarcoma and can contribute to better management of the condition.

## Data Availability

The generated and analyzed datasets of the current research are available freely in the TARGET database (https://ocg.cancer.gov/programs/target) and GEO database (https://www.ncbi.nlm.nih.gov/gds).

## Supplementary Materials

Supplementary 1 Table S1: sample clinical features.

Supplementary 2 Table S2: summary of GSEA results.

Supplementary 3 Figure S1: list of the top 150 hypoxia-associated genes. The size and color of the genes in the word cloud reflect how closely related they are to one another.
