# Supplementary material for: A Hypoxia Gene-Based Signature to Predict the Survival and Affect the Tumor Immune Microenvironment of Osteosarcoma in Children
Source: J Immunol Res. 2021 Jul 15;2021:5523832. doi: 10.1155/2021/5523832 (PMC8299210; doi:10.1155/2021/5523832)
Supplement: Supplementary 1 — Table S1: sample clinical features. [file 5523832.f1.docx]

**Table S1** Sample clinical features

| **Clinical features** | **TARGET** | **GSE21257** |
| --- | --- | --- |
| **Status** |  |  |
| Alive | 55 | 30 |
| Dead | 29 | 23 |
| **Gender** |  |  |
| Male | 47 | 34 |
| Female | 37 | 19 |
| **Metastatic** |  |  |
| Yes | 21 | 34 |
| No | 63 | 19 |
| **Age** |  |  |
| ≤15 | 46 | 21 |
| ≥15 | 38 | 32 |
