# Supplementary material for: A Hypoxia Gene-Based Signature to Predict the Survival and Affect the Tumor Immune Microenvironment of Osteosarcoma in Children
Source: J Immunol Res. 2021 Jul 15;2021:5523832. doi: 10.1155/2021/5523832 (PMC8299210; doi:10.1155/2021/5523832)
Supplement: Supplementary 2 — Table S2: summary of GSEA results. [file 5523832.f2.docx]

Table S2. Summary of GSEA results (top 10)

| GS follow link to MSigDB | ES | NES | NOM p-val | FDR q-val |
| --- | --- | --- | --- | --- |
| KEGG_HEDGEHOG_SIGNALING_PATHWAY | 0.60 | 1.85 | 0.002 | 0.519 |
| KEGG_NITROGEN_METABOLISM | 0.63 | 1.79 | 0.017 | 0.387 |
| KEGG_ABC_TRANSPORTERS | 0.59 | 1.68 | 0.032 | 0.385 |
| KEGG_RIBOSOME | 0.75 | 1.74 | 0.058 | 0.366 |
| KEGG_TERPENOID_BACKBONE_BIOSYNTHESIS | 0.53 | 1.45 | 0.115 | 1.000 |
| KEGG_GLYCINE_SERINE_AND_THREONINE_METABOLISM | 0.40 | 1.43 | 0.077 | 0.938 |
| KEGG_TYROSINE_METABOLISM | 0.38 | 1.40 | 0.091 | 0.917 |
| KEGG_BIOSYNTHESIS_OF_UNSATURATED_FATTY_ACID | 0.43 | 1.30 | 0.190 | 1.000 |
| KEGG_PENTOSE_AND_GLUCURONATE_INTERCONVERSIONS | 0.48 | 1.29 | 0.196 | 1.000 |
| KEGG_MTOR_SIGNALING_PATHWAY | 0.31 | 1.19 | 0.229 | 1.000 |
