# Supplementary figures and images for: A Hypoxia Gene-Based Signature to Predict the Survival and Affect the Tumor Immune Microenvironment of Osteosarcoma in Children
Source: J Immunol Res. 2021 Jul 15;2021:5523832. doi: 10.1155/2021/5523832 (PMC8299210; doi:10.1155/2021/5523832)

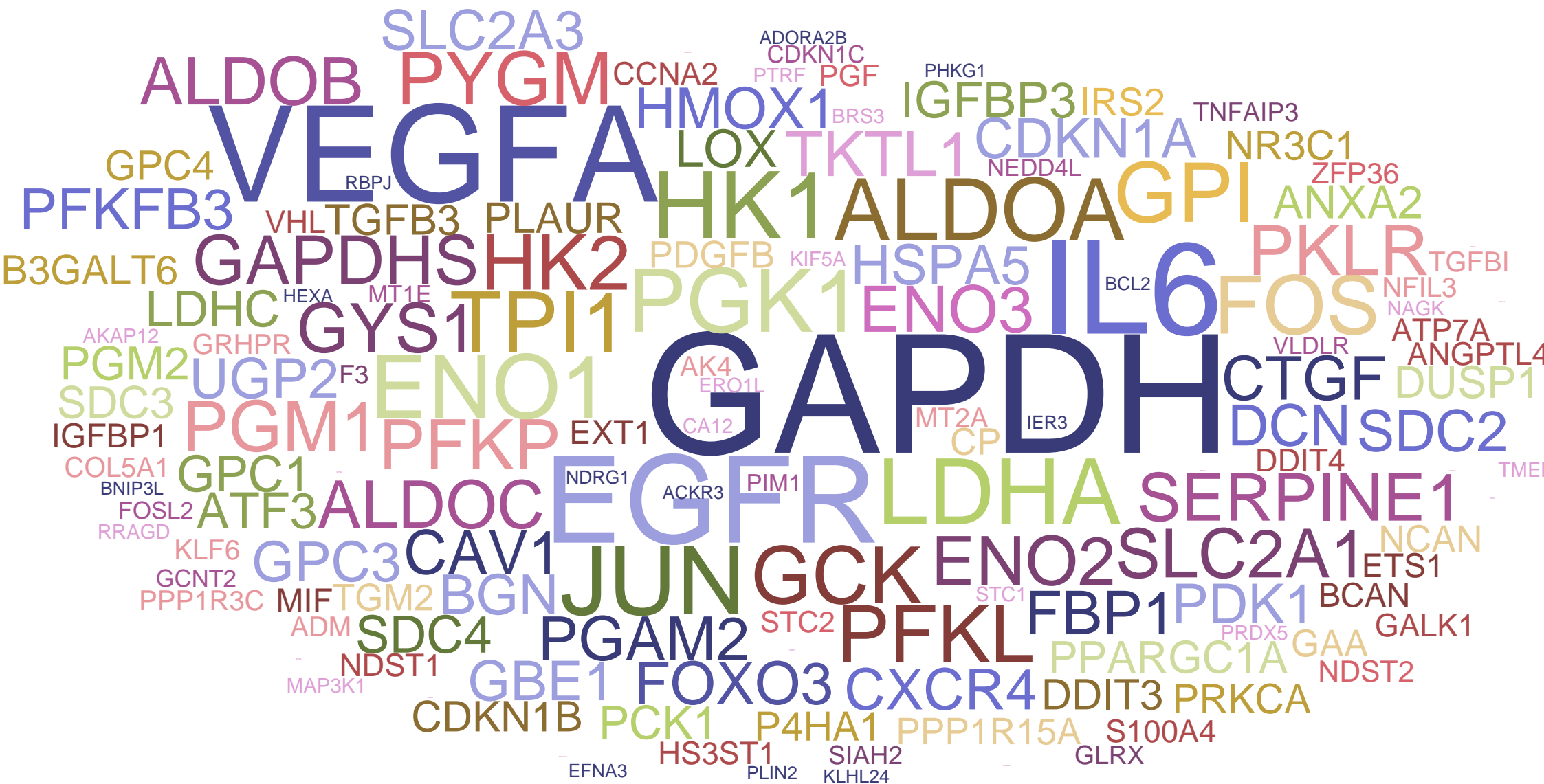

Supplement: Supplementary 3 — Figure S1: list of the top 150 hypoxia-associated genes. The size and color of the genes in the word cloud reflect how closely related they are to one another. [file 5523832.f3.pdf]
